# Evaluation of micro-GPS receivers for tracking small-bodied mammals

**DOI:** 10.1371/journal.pone.0173185

**Published:** 2017-03-16

**Authors:** Laura A. McMahon, Janet L. Rachlow, Lisa A. Shipley, Jennifer S. Forbey, Timothy R. Johnson, Peter J. Olsoy

**Affiliations:** 1 Department of Fish and Wildlife Sciences, University of Idaho, Moscow, Idaho, United States of America; 2 School of the Environment, Washington State University, Pullman, Washington, United States of America; 3 Department of Biological Sciences, Boise State University, Boise, Idaho, United States of America; 4 Department of Statistical Science, University of Idaho, Moscow, Idaho, United States of America; University of Sydney, AUSTRALIA

## Abstract

GPS telemetry markedly enhances the temporal and spatial resolution of animal location data, and recent advances in micro-GPS receivers permit their deployment on small mammals. One such technological advance, snapshot technology, allows for improved battery life by reducing the time to first fix via postponing recovery of satellite ephemeris (satellite location) data and processing of locations. However, no previous work has employed snapshot technology for small, terrestrial mammals. We evaluated performance of two types of micro-GPS (< 20 g) receivers (traditional and snapshot) on a small, semi-fossorial lagomorph, the pygmy rabbit (*Brachylagus idahoensis*), to understand how GPS errors might influence fine-scale assessments of space use and habitat selection. During stationary tests, microtopography (i.e., burrows) and satellite geometry had the largest influence on GPS fix success rate (FSR) and location error (LE). There was no difference between FSR while animals wore the GPS collars above ground (determined via light sensors) and FSR generated during stationary, above-ground trials, suggesting that animal behavior other than burrowing did not markedly influence micro-GPS errors. In our study, traditional micro-GPS receivers demonstrated similar FSR and LE to snapshot receivers, however, snapshot receivers operated inconsistently due to battery and software failures. In contrast, the initial traditional receivers deployed on animals experienced some breakages, but a modified collar design consistently functioned as expected. If such problems were resolved, snapshot technology could reduce the tradeoff between fix interval and battery life that occurs with traditional micro-GPS receivers. Our results suggest that micro-GPS receivers are capable of addressing questions about space use and resource selection by small mammals, but that additional techniques might be needed to identify use of habitat structures (e.g., burrows, tree cavities, rock crevices) that could affect micro-GPS performance and bias study results.

## Introduction

GPS telemetry markedly enhances the temporal and spatial resolutions of animal location data. However, until recently, the size and weight of GPS receivers restricted their applications on small species. Although > 90% of mammals weigh < 5 kg (Fleming 1979), relatively few small mammals have been tracked using GPS telemetry. Advances in GPS technologies and smaller, more powerful batteries provide increasing opportunities for collecting GPS locations for small species. Comprehensive studies are needed to evaluate under what conditions micro-GPS receivers can provide the high precision location data necessary to address questions about movement and resource selection at relevant scales for small, mammalian species.

Advances in GPS technology and miniaturization of electronics and batteries now permit GPS deployment on small mammals weighing < 1 kg. To decrease size and weight, lightweight traditional GPS receivers often require small batteries, which leads to a trade-off between the number of locations generated and duration of battery life. Traditional receivers calculate positions in real time, although the amount of time necessary to acquire a location estimate varies (Moriarty and Epps 2015). Increasing the sampling rate to acquire more frequent locations will deplete batteries more quickly, thereby reducing the duration of data collection. Recent applications of traditional GPS telemetry for mammals <1 kg have faced such tradeoffs (e.g., Egyptian fruit bats, *Rousettus aegyptiacus* [1]; Madagascar flying foxes, *Pteropus rufus* [2]; gray squirrels, *Sciurus carolinensis* [3]; European hedgehogs, *Erinaceus europaeus* [4]; and snowshoe hares, *Lepus americanus* [5]). Snapshot technology, which was originally developed for marine species that only briefly surface above water [6], maximizes the life of small and lightweight batteries, thereby minimizing the tradeoff between battery life and frequency of GPS locations. To accomplish this, the snapshot receiver digitizes and stores raw data (satellite identification and timestamps) from satellite signals in <1 s, after which the GPS shuts down until its next scheduled fix [7]. The receiver postpones the recovery of ephemeris data (i.e., precise satellite information) and calculation of receiver locations until after recovery of the receiver, when the raw data, in conjunction with post-processing software and downloaded data, are used to estimate GPS locations in a similar manner to traditional GPS receivers.

With technological advances, manufacturers are marketing smaller GPS telemetry receivers for wildlife research. However, to the best of our knowledge, limited information is available about the magnitude of errors associated with these micro-GPS receivers in relation to habitat features. No published research has evaluated snapshot GPS collection for small mammals, and we are aware of only two published studies [1,4] evaluating the performance of micro-GPS receivers (< 20 g) using traditional GPS collection for small mammals. Neither of these studies quantified the influence of habitat characteristics (e.g., vegetation cover or topography) or satellite geometry and availability on successful GPS location estimation and accuracy.

GPS errors typically are associated with environmental characteristics (e.g., terrain, vegetation), satellite acquisition (e.g., number of satellites detected, satellite geometry), and animal behavior. To estimate a location, a GPS receiver must receive satellite signals to triangulate its position, and this reliance on satellites can lead to two common types of performance errors: 1) a reduction in fix success rate (FSR; the number of successful fix attempts divided by the total attempted fixes) when the GPS cannot acquire signals from enough satellites to generate a location estimate; and 2) location errors (LE; the Euclidian distance between each GPS-generated location and the true location), which occur when a GPS receiver inaccurately triangulates a location [8]. Rugged terrain often is associated with poor GPS performance because topography blocks satellite reception, leading to low FSR [9–12]. Vegetation characteristics, such as canopy closure, can increase LE through multipath error, a result of satellite signals reflecting off of the canopy and woody material [13,14]. The magnitude of location error also is affected by satellite geometry and the number of satellites used to determine a specific location. The GPS receiver computes locations based on the angles of satellites (triangulation), and successful locations are classified as 2-dimensional (2-D) if the GPS exploited 3 satellites or 3-dimensional (3-D) if the GPS used >3 satellites for these calculations. Location error decreases as the number of satellites used to generate a location increases; for that reason, 3-D fixes are most often associated with smaller location errors [11]. Finally, error is introduced when the receiver is worn by an animal. Behavior of the animal (e.g., foraging, resting, burrowing) can reduce FSR or increase LE by altering the angle of the GPS antenna [15] or as a result of use of habitats that are suboptimal for satellite acquisition, such as dense cover, burrows, or tree cavities [16,17]. Location error can result in biases in research studies by masking habitat selection at fine-scales, especially in heterogeneous landscapes when LE is greater than the selected patch sizes [8,18]. Likewise, low FSR could bias habitat selection by underrepresenting use of habitat types where the GPS has a lower probability of obtaining a successful location estimate [8].

To fill the gap in understanding of micro-GPS performance and its potential to track movement by small mammals, we evaluated performance of two types of micro-GPS telemetry receivers (traditional and snapshot) using stationary tests and field trials on a small (375–450 g), semi-fossorial lagomorph, the pygmy rabbit (*Brachylagus idahoensis*), which inhabits sagebrush steppe environments in the western USA [19]. First, we hypothesized that the two types of receivers would have similar FSR, but that traditional micro-GPS would have a lower LE because of extended time to search for satellites. Second, we hypothesized that shrub cover would influence performance of the GPS devices by interfering with the angles of satellite detection. We predicted that shrub cover would be positively correlated with LE and negatively correlated with FSR. Third, we hypothesized that burrowing behavior of rabbits would reduce micro-GPS telemetry performance by obstructing satellite reception, and we expected lower FSR and greater LE when micro-GPS receivers were in burrows of pygmy rabbits. Finally, because previous research has documented that animal behavior influences GPS performance [20,21], we used light sensors paired with GPS collars to identify and screen out location attempts when animals were in burrows to facilitate an evaluation of the influence of behavior on fix success while animals were above ground. We expected to document lower FSR with both technologies from receivers deployed on animals above ground in the field in comparison to results from above-ground stationary tests. We compare our results to data reported from GPS telemetry on large-bodied mammalian species to provide context for considering the scaling of errors with micro-GPS receivers on small species.

## Methods

### Study area

We conducted this research at Cedar Gulch (44° 41' N, 113° 17' W), located within the Lemhi Valley in east-central Idaho, USA. The Lemhi Valley runs parallel to the Montana border, and is bounded by the Beaverhead Mountains to the east and the Lemhi Mountain Range to the west. The Cedar Gulch study site has limited topography (elevation ranged between 1,880 and 1,925 m), with higher elevations occurring in the northern region.

The study site encompassed approximately 100 ha of continuous sagebrush-steppe habitat characterized by distinct dome-like mounds of sediments (i.e., mima mounds). At the Cedar Gulch site, the mean diameter of mima mounds was 10.6 m [22] and mounds were separated by 30–50 m. Wyoming big sagebrush (Artemisia tridentata subsp. wyomingensis) shrubs occurred predominantly clumped on these mounds and also dominated off-mound areas along with low densities of black sagebrush (A. nova) and three-tip sagebrush (A. tripartite). Compared to the off-mound matrix, mima mounds supported taller and denser shrubs (shrub height = 0.5–1.0 m; [22]), which provided more potential concealment cover for wildlife such as pygmy rabbits than off mounds, creating a heterogeneous landscape lacking in trees and large woody obstructions [23]. At this site, pygmy rabbit burrows occurred almost exclusively on mima mounds. Rabbit burrow systems are simple tunnels consisting of one or more entrances, with the size of the entrance openings varying from 6 to 31 cm [19].

### Stationary tests

**Fig 1:**
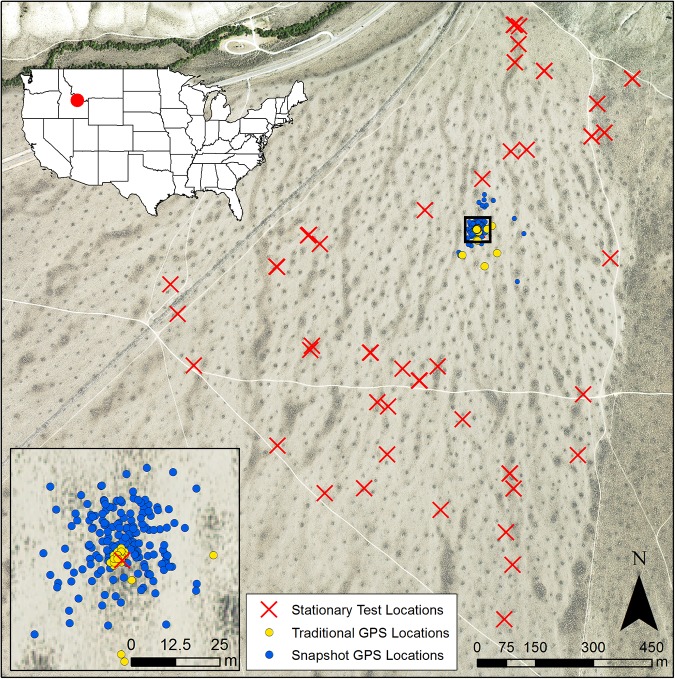
GPS data collection by GPS receivers at a stationary test location at Cedar Gulch. GPS data collection by snapshot (blue circles) and traditional (yellow circles) at one stationary test location. Stationary test locations (red Xs) were distributed throughout the Cedar Gulch study site. Darker regions in the aerial imagery acquired from the National Agriculture Imagery Program (NAIP) display areas of denser shrub cover.

We evaluated performance of two types of GPS technology, snapshot and traditional, in stationary tests at randomly located sites throughout our study area. We stratified Cedar Gulch into two strata representing areas on and off of mima mounds and used a stratified random sampling design to identify 20 on- and 20 off-mound test locations (n = 40) for placement of GPS receivers (Fig 1). To facilitate evaluation of the influence of burrows on GPS performance, we constrained our on-mound test sites to mima mounds with rabbit burrows (n = 195) using a data layer of pygmy rabbit burrow locations within Cedar Gulch. To evaluate the effects of burrowing behavior (i.e., use of underground burrows vs. above-ground locations), we paired 10 of the on-mound locations with a GPS receiver placed in a randomly selected burrow tunnel at a consistent distance of 0.25 m from the burrow entrance. At 0.25 m, the device is completely underground, however, it is near enough to the burrow entrance that it is possible to detect some satellites through the opening. The paired on-mound test site was located on the ground surface directly above the underground receiver. With this configuration, both receivers were located at the same coordinates with the only difference being the separation between above and below ground. All test locations were marked at < 3-cm accuracy using a survey-grade GPS unit (Topcon HiPer V, Topcon Positioning Systems, CA, United States, https://www.topconpositioning.com) to provide known references against which to compare GPS-estimated locations [24].

We deployed both types of GPS receivers on collars weighing < 15 g. Test receivers were attached with plastic zip-ties to wooden stakes at 8 cm above the ground (approximate height of a GPS collar worn by a pygmy rabbit) for approximately 48-hr periods and were programmed to record a location every 15 min (Table 1). At a subset of locations, we deployed two receivers simultaneously to evaluate variability in performance between receivers. We tested 18 G10 UltraLITE GPS Loggers (snapshot receivers; Advanced Telemetry Systems, MN, United States, http://atstrack.com) during summer 2015 and 12 FLR V GM502030 Loggers (traditional receivers; Telemetry Solutions, CA, United States, http://www.telemetrysolutions.com) during summer 2016. Snapshot receivers were programmed at the highest sensitivity, allowing the receivers to detect satellites for 512 ms before shutting down. Traditional receivers were programmed to collect satellite information for a maximum of 90 s. Snapshot receivers logged information regarding date, time, latitude, longitude, number of satellites, and dilution of precision (DOP) values including horizontal (HDOP), vertical (VDOP), and positional (PDOP) [25]. Traditional receivers recorded date, time, northing, easting, time to fix (TTF) in seconds, type of fix (i.e., 3-D and 2-D), HDOP, and battery voltage. Dilution of precision values measure satellite geometry. Wider spacing of satellites (i.e., more optimal geometry) results in lower DOP (e.g., HDOP) values, which may indicate greater locational precision (i.e., lower LE) [26,27].

**Table 1:** GPS evaluations (i.e., stationary tests and field deployments) conducted at Cedar Gulch during 2015–2016.

Experiment	GPS Type	Year	Sample Size
**Stationary Tests**			
	Snapshot	2015 (W, S)	87
	Traditional	2016 (W, S)	46
**Field Deployments**			
	Snapshot	2015 (W, S)	29
	Traditional	2016 (W, S)	44
	Snapshot + Light Sensor	2015 (S)	8
	Traditional + Light Sensor	2016 (W)	10

W, Winter; S, Summer.

Stationary tests allowed us to evaluate the influence of a suite of environmental characteristics (shrub canopy, height, and burrow cover) on performance of GPS receivers [24]. In the field, we quantified vegetation closure measured as the proportion of sky obscured by vegetation or woody material at an approximate 1-m scale using hemispherical photography. Photographs were captured using a smartphone camera equipped with a fish-eye lens, leveled at 8 cm and directed upwards from the test location [24]. Using the program ‘SamplePoint’ [28], we overlaid a grid with 100 intersections onto each photograph, and classified each intersection within the image as either vegetation or sky. We used the proportion of intersections classified as vegetation as an estimate of vegetation closure. To estimate mean shrub height, we recorded the heights of all shrubs > 8 cm within 1 m of the test location to produce a mean value. We evaluated other shrub characteristics at two extents surrounding test collar locations (6- and 12-m radius buffers) using a shrub data layer classified from 2.74-cm resolution imagery collected from unmanned aerial vehicles. To estimate shrub canopy in the buffered regions surrounding each test location, the number of pixels classified as shrub were summed and divided by the total number of pixels. Because our study site was open and relatively flat with limited terrain obstruction, we did not include variables such as available sky, elevation, or slope in our analyses.

We evaluated errors for each micro-GPS receiver type by calculating FSR and LE [13]. Because preliminary analysis of snapshot receivers indicated more variable performance at the start of each stationary test, we used segmented regression to identify a break point in the data that represented the time at which location error stabilized. We excluded the more variable initial fixes from snapshot model development to assess the influence of burrow cover and habitat characteristics. Additionally, we examined if FSR and LE varied by receiver type or burrow cover (i.e., in or out of a burrow). We modeled FSR and LE separately for both receiver types to identify variables that influenced performance. Models were created based on a priori hypotheses, and we included random effects for receiver and site to account for non-independence among location attempts within devices and/or sites [10].

To assess strength of evidence, we evaluated all models using the corrected Akaike Information Criterion for small sample sizes (AICc) [29]. All statistical analyses were conducted using R 3.2.3 [30]. Mixed-effects modeling was conducted in the lme4 package [31]. We removed models with uninformative parameters (i.e., log-likelihood, LL, was nearly unchanged with the addition of variables) from reported model sets and recalculated model weight [32]. For mixed-effects model results, we inferred significance of variables if the 85% confidence intervals for the odds ratios did not overlap one and parameter estimates did not overlap zero [32].

We used mixed-effects logistic regression to model the influence of burrow cover and shrub characteristics on FSR to evaluate the probability of obtaining a successful fix (2-D and 3-D). We fit nine models for each technology type that included an intercept-only null model. Predictor variables included burrow cover (in or out), vegetation closure, average shrub height within 1 m of the test locations, and shrub canopy cover in each of the two buffers (6 and 12 m). For data derived from snapshot receivers, we included interactions between burrow cover and vegetation closure, shrub height (1 m), and shrub canopy (6 m). Because of small sample sizes, we could not include interactions among variables in our traditional GPS model set.

We modeled LE using mixed-effects linear regression with a log transformation of the response variable to better meet the assumptions of the model [11]. We included the same predictors used to model FSR, with the addition of technical variables (number of satellites, HDOP, and TTF). The number of satellites and HDOP were highly correlated, and therefore, were not included in the same models. Time to fix (TTF) was included in modeling LE data from traditional GPS receivers, but was not included in modeling performance of snapshot technology because the snapshot receivers were set to turn on for a standard period of time. We evaluated 19 candidate models with the snapshot GPS data and 20 candidate models with traditional GPS data.

Initial analyses of FSR and LE with respect to burrow, shrub characteristics, and satellite availability indicated an overwhelmingly strong influence of burrow on error. To determine if the effect of burrow cover masked the influences of vegetation and satellite characteristics, we performed a post-hoc analysis by repeating the logistic and linear mixed-effects models with only data collected from above-ground collected receivers. We revised the original model sets by removing the burrow cover variable.

After completing stationary tests, we noted marked variation in performance among individual receivers of both technologies. Consequently, we conducted an additional test of consistency by operating receivers simultaneously in open habitat. Receivers were placed on wooden stakes separated by 1 m in a 3x4 m grid pattern. We programmed the 12 traditional receivers to collect 1 location every 15 min for 48 hr, and conducted a one-way analysis of variance (ANOVA) on the log of LE. We did not conduct a similar consistency test of snapshot receivers because they did not function reliably.

### Field performance trials

**Fig 2:**
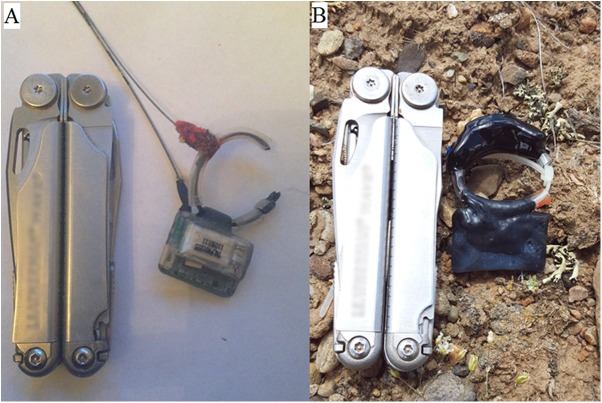
Micro-GPS receivers deployed in this study. (A) G10 UltraLITE GPS Logger (snapshot GPS receivers) weighed 11 g. (B) FLR V GM502030 GPS Logger (traditional GPS receivers) weighed 14–16 g.

To evaluate FSR of GPS receivers while deployed on animals, we fitted adult pygmy rabbits weighing > 415 g with GPS receivers attached to a collar. This weight threshold ensured that the collar was ≤ 5% of body mass [33,34]. Animals were trapped using Tomahawk live traps (Wisconsin, United States) and fitted with GPS collars (snapshot receivers– 10.6 g or traditional receivers– 13.8–15.1 g; Fig 2) with an attached very high frequency (VHF) transmitter (BD-2–1.6 g, Holohil Systems Ltd., Ontario, Canada) during winter (January-March) and summer (June-August) of 2015 and 2016 (Table 1). To maximize battery life, snapshot receivers were set to collect one location every 15 minutes and traditional receivers were set to collect one location every hour. Battery life at these respective fix intervals was estimated at 118 days for snapshot receivers and 9–13 days for traditional receivers. The GPS collars remained on individuals for 2–6 weeks, after which the rabbits were trapped to remove the collar and download data. All methods used in this study were approved by the University of Idaho Animal Care and Use Committee (Protocol #2015–12) and complied with the guidelines for use of wild mammals in research published by the American Society of Mammalogists [33]. Animal capture was approved by the Idaho Department of Fish and Game (scientific collecting permit #010813). Because our study was conducted on public land managed by the Bureau of Land Management on a species not currently listed as threatened or endangered, additional permissions were not required.

To determine whether failed fixes were influenced by burrow use or other animal behaviors, we attached 1-g light sensors (Intigeo-C65, Migrate Technology, Cambridge, United Kingdom) that logged the highest light levels (lux) every 5 minutes to the collars of a subset of the rabbits. During summer 2015, light sensors were paired with snapshot receivers, and in winter 2016, light sensors were paired with traditional receivers (Table 1). Because we could only determine burrow use using the light sensor during daylight hours, we focused our analysis on GPS fix-lux pairings between sunrise and sunset. A GPS fix-lux pairing occurred when the GPS location and light reading were obtained within 5 minutes of each other. Because our sampling unit was an individual rabbit, we used a linear mixed-effect model with a random intercept for individual rabbit to make inferences concerning the overall (population mean) lux levels between successfully acquired and missed fixes. To identify lux levels that would indicate when rabbits were underground, we tested light sensor performance at two depths (20 and 30 cm) within burrows and also placed one sensor outside of the burrows, under a shrub. We conducted these trials at two burrow locations during winter and summer. We used lux levels from within the burrows to identify a 95th-percentile threshold for each season to classify each GPS fix-lux pairing as above or below ground. With this method, we aimed to minimize contamination of above-ground GPS fixes with underground fixes to facilitate evaluation of the influence of behavior on fix success while the animal was above ground.

## Results

### Stationary performance

Receivers were placed at stationary test locations for an average of 48 hr (range = 46–48) during which we evaluated both FSR and LE relative to habitat parameters. Vegetation closure at these sites ranged from 0–81%. Between June and October 2015, we attempted 126 stationary tests using 18 snapshot receivers, however, the GPS receivers operated for the full 48-hr period during only 87 stationary tests at 37 test sites (31% failure rate). During July and October 2016, we completed 46 stationary tests at the same 37 locations using 12 traditional GPS receivers, all of which successfully recorded locations. Although 10 burrow sites were selected for testing, one burrow collapsed during the first year of sampling and was removed from the analyses, and another burrow was modified by rabbits between the first and second year, resulting in additional entrances. We removed that test site from analyses because the receiver could not be placed 25 cm into a burrow without being < 25 cm from another burrow entrance. As a result, of the 87 snapshot receiver trials, 13 tests were conducted underground in burrows at 9 sites, and of the 48 traditional GPS trials, 7 tests were conducted underground in burrows at 7 sites. Although 8 possible burrow sites were available for traditional tests, only 7 collected usable data because the receiver at the 8^th^ burrow site was visibly disturbed and repositioned by an animal over the course of the 48-hr trial and was removed from the analysis.

**Fig 3:**
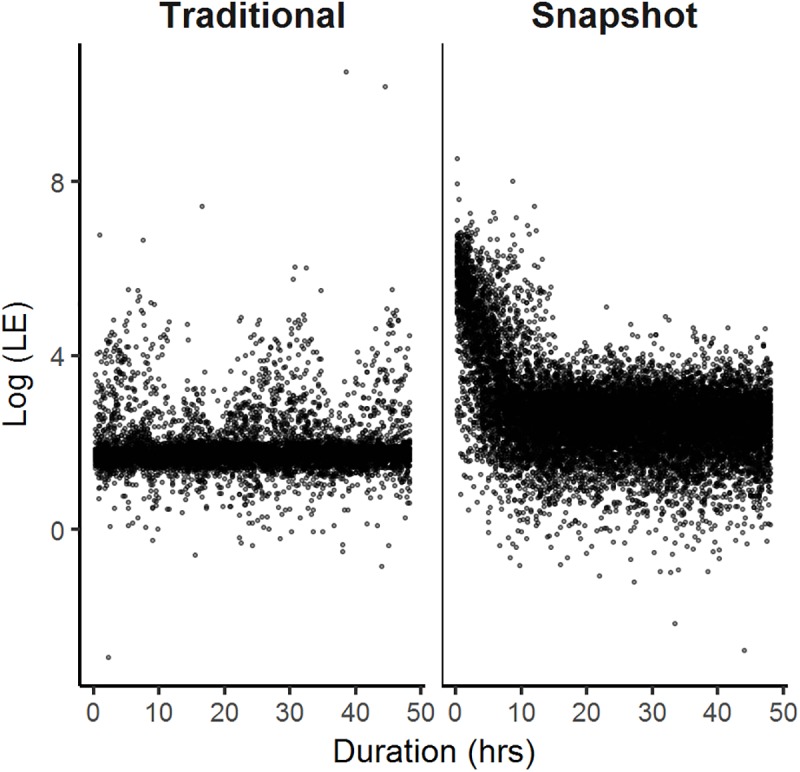
Relationship between duration (hr) from the start of the stationary tests and micro-GPS accuracy (log of location error [LE]). Traditional micro-GPS receivers (left) displayed consistent performance across the entire 0range of duration time, whereas snapshot micro-GPS receivers (right) generated large LE values at the start of each stationary test before leveling to a more consistent performance.

Technological differences between snapshot and traditional receivers were evident by the types of fixes collected and the variation in data quality at the start of each stationary test. Snapshot receivers only acquired 3-D fixes (> 3 satellites), whereas traditional micro-GPS receivers collected both 3-D and 2-D (3 satellites) fixes. The start of each snapshot stationary test showed greater variability in location errors, and results of the segmented regression analysis suggested that snapshot receivers stabilized and collected more consistent and accurate data after 9.2 ± 0.12 hr (Fig 3). Because of this, we screened the first 37 locations from each snapshot test. No breakpoint was needed for the data recorded by traditional micro-GPS receivers because the data displayed consistent performance across the entire 48-hr testing period (Fig 3).

**Fig 4:**
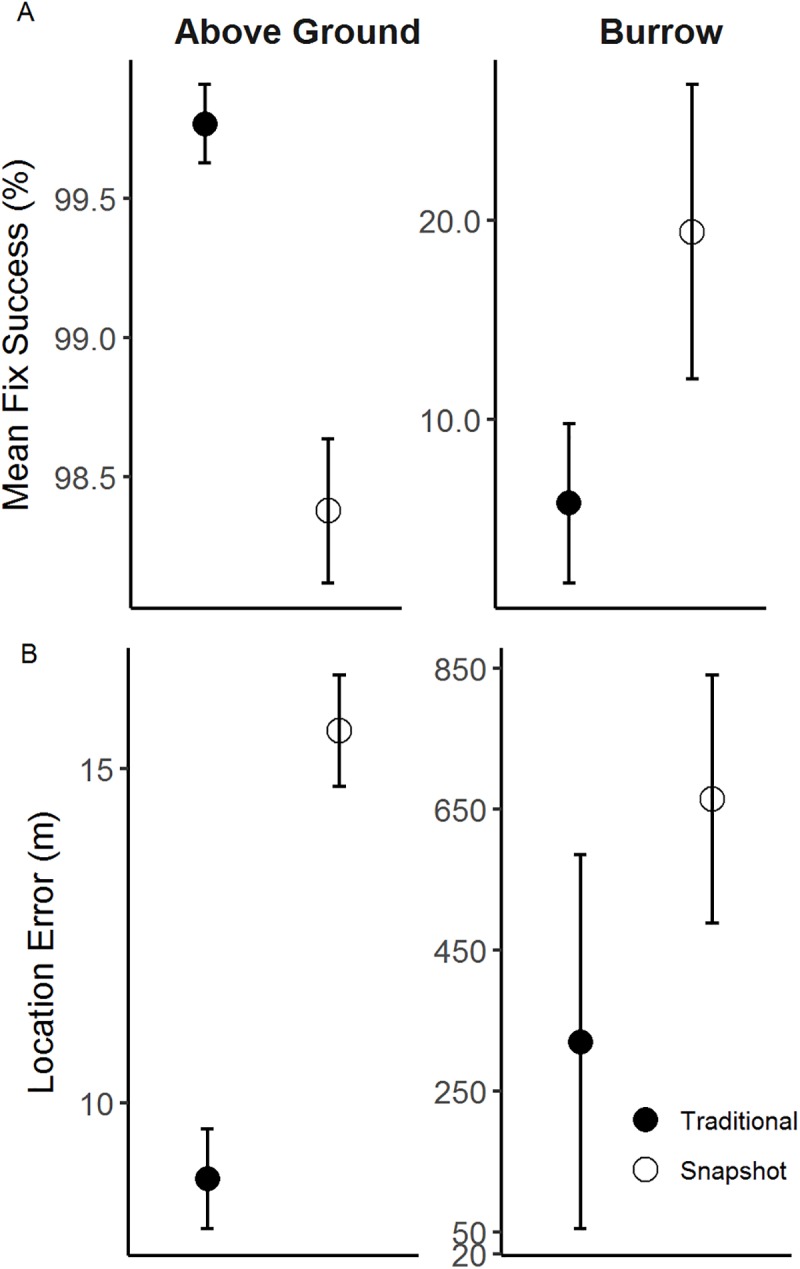
Performance of micro-GPS receiver during stationary tests for locations in burrows and above ground. Mean values (±SE) of (A) fix success rate (%) and (B) location error (m) for traditional and snapshot micro-GPS receivers placed on the ground surface and in burrows at a depth of 25 cm.

As expected, micro-GPS performance in our stationary tests was influenced by both burrow cover and receiver type. Above ground, overall FSR was higher (F_1, 130_ = 1496.00, p < 0.001) and LE values were about 30 times smaller (F_1, 123_ = 376.49, p < 0.001) than receivers placed in burrows (Fig 4). Additionally, above-ground receivers obtained locations using more satellites ($\bar{x}=8.1\pm 0.07$) with lower HDOP values ($\bar{x}=4.4\pm 0.02$) than receivers below ground (satellites: $\bar{x}=6.2\pm 0.01$; HDOP: $\bar{x}=9.9\pm 0.19$). We observed a significant interaction between receiver type and burrow cover on FSR (F_1,128_ = 12.17, p < 0.001) and LE (F_1, 121_ = 20.36, p < 0.001). Whereas receiver type within burrows did not significantly influence FSR (F_1,18_ = 1.66, p = 0.21), it did significantly influence LE (F_1, 11_ = 5.89, p = 0.03). Below ground, traditional micro-GPS receivers obtained locations with lower LE values ($\bar{x}=320.2\pm 264.56\;m$) than snapshot receivers ($\bar{x}=663.7\pm 175.60$ m). In addition, traditional receivers placed above ground acquired significantly higher rates of successful locations (F_1,110_ = 13.53, p < 0.001) with lower LE values (F_1, 110_ = 369.82, p < 0.001) than snapshot receivers. Location errors for snapshot receivers above ground ($\bar{x}=15.5\pm 0.27\;m$) were almost double that of traditional receivers ($\bar{x}=8.9\pm 0.33\;m$). Snapshot micro-GPS receivers detected more satellites regardless of placement in or out of a burrow ($\bar{x}=8.7\pm 0.01$) than traditional receivers ($\bar{x}=7.0\pm 0.02$), although mean HDOP values were lower in traditional ($\bar{x}=1.6\pm 0.01$) than snapshot receivers ($\bar{x}=6.3\pm 0.02$), suggesting that traditional receivers were using satellites with more optimal satellite geometry.

Burrow cover and vegetation structure influenced FSR for both types of micro-GPS receivers in stationary tests. For snapshot GPS receivers, the top-ranked model contained 100% of the model weight (Table 2), and suggested that for receivers above ground, a 5% increase in vegetation closure resulted in a 9% increase in FSR. For receivers in burrows, a 5% increase in vegetation closure decreased the probability of a successful fix by 41%. Model selection for traditional GPS receivers revealed five models with 95% of the cumulative model weight, with the top two models receiving a cumulative weight of 52% (Table 2). The top-ranked model suggested that the probability of a successful fix decreased by nearly 100% when below ground and decreased by 38% for every 5% increase in surrounding canopy cover, and the second-ranked model included the effects of burrow cover and shrub height (Table 2). That model also suggested that the probability of a successful fix decreased by nearly 100% when below ground and in addition, fix success decreased by 4% for every 1-cm increase in shrub height. Although no single model received overwhelming support, the effect of burrow cover was present in all models indicating that burrows significantly influenced the odds of obtaining a successful fix, whereas surrounding canopy cover, shrub height and other vegetation characteristics (i.e., vegetation closure, surrounding shrub canopy 12-m) had only moderate, non-significant additive influences on fix success.

**Table 2:** Models explaining fix success rate (FSR) of snapshot and traditional micro-GPS receivers at 37 stationary test sites.

Micro-GPS	Model	K	AICc	ΔAICc	Wt	LL	Rank
Snapshot							
	Burrow + Vegetation Closure–Burrow X Vegetation Closure*	6	3106.63	0.00	1.00	-1547.3	1
Traditional							
	- Burrow* - Surrounding (6 m) Canopy	5	641.41	0.00	0.26	-315.7	1
	- Burrow* - Shrub Height	4	641.49	0.08	0.25	-315.8	2
	- Burrow*	5	641.52	0.10	0.25	-316.8	3
	- Burrow* - Vegetation Closure	5	642.97	1.55	0.12	-316.5	4
	- Burrow* - Surrounding (12 m) Canopy	5	643.15	1.73	0.11	-316.6	5

K, the number of parameters estimated; ΔAICc, the change in AICc; Wt, model weight; LL, log-likelihood.

*Significant at the 85% confidence level

(+) positive influence of covariate and (-) negative influence of covariate on FSR.

During stationary tests, LE was influenced by burrow cover, and to a lesser extent, satellite geometry and vegetation. For snapshot GPS receivers, the top model describing the LE had 99% of the model weight and included burrow cover, vegetation closure, their interaction, and HDOP. The interaction (vegetation closure * burrow cover) and HDOP were significant at the 85% confidence level (Table 3). Values for LE increased with greater vegetation closure and compounded error for receivers within burrows to augment the magnitude of LE. Finally, satellite geometry (HDOP) was positively correlated with location error. Unlike snapshot receivers, there were four plausible models describing LE for traditional receivers, although the top-ranked model received 40% of the model weight. The top-ranked model included burrow cover and HDOP, whereas the other three competing models included the same two variables with the addition of shrub characteristics (Table 3). Similar to snapshot GPS technology, traditional receivers were influenced by being underground, with a larger magnitude of error observed for receivers below ground. Accuracy of traditional GPS receivers declined with larger HDOP values, and that trend was evident in all competing models. The shrub characteristics in the top models had parameter estimates with 85% confidence intervals overlapping 0, suggesting limited influence on magnitude of LE for traditional GPS telemetry.

**Table 3:** Models explaining location error (LE) of snapshot and traditional micro-GPS receivers at 37 test sites.

Micro-GPS	Model	K	AICc	ΔAICc	Wt	LL	Rank
**Snapshot**							
	Burrow + HDOP* + Vegetation Closure + Burrow X Vegetation Closure*	8	26288.8	0	0.99	-13136.0	1
**Traditional**							
	Burrow* + HDOP*	6	12876.5	0	0.40	-6432.3	1
** **	Burrow* + HDOP* - Vegetation Closure	7	12877.3	0.77	0.27	-6431.7	2
	Burrow* + HDOP* - Surrounding (6m) Canopy	7	12878.1	1.55	0.18	-6432.0	3
** **	Burrow* + HDOP* - Shrub Height	7	12878.4	1.92	0.15	-6432.2	4

K, the number of parameters estimated; ΔAICc, the change in AICc; Wt, model weight; LL, log-likelihood; Rank, order of models within the top model set.

*Significant at the 85% confidence level; (+) positive influence of covariate and (-) negative influence of covariate on FSR.

Constraining the analysis to data collected from above-ground trials, we observed strong influences of vegetation and satellite availability on LE and FSR for both snapshot and traditional receivers, suggesting that the effect of burrow cover masked the influence of additional variables. For snapshot receivers, the intercept only model for FSR received 35% of the model weight, suggesting vegetation had minimal influence on FSR; however, the top model describing LE had 85% of the model weight and included two significant fixed effects, surrounding shrub canopy (6 m) and the number of satellites. That model suggested that LE values increased by 2.6% for every 5% increase in canopy cover and decreased by 5.0% for every additional satellite used to calculate the location. Unlike snapshot receivers, there were four plausible models describing FSR for traditional receivers, although the two top-ranked models received 81% of the cumulative model weight. The top-ranked model included shrub height, whereas the second-ranked model included only vegetation closure. Both variables significantly reduced the probability of obtaining a fix. Finally, post-hoc model selection for LE of traditional GPS receivers revealed four models with 95% of the cumulative model weight. The top-ranked model received 58% of the model weight and included a significant negative effect of shrub height on LE and a positive relationship between LE and HDOP. The 3 additional models in the 95% confidence set also included a significant positive relationship between HDOP and LE. The effect of HDOP was present in all models indicating that HDOP significantly influenced LE, whereas surrounding canopy cover and vegetation closure had only weak, negative correlations with location error.

The consistency test indicated similar probabilities of obtaining locations across traditional micro-GPS receivers, but relatively large variation in accuracy of the locations. FSR was similarly high (mean = 99.0%, range = 97–100%) across all receivers, however, mean LE ranged between 4.1 and 17.1 m. Analysis of variance indicated significant differences in LE among the receivers (F_11,2234_ = 12.4; p < 0.001).

### Field performance trials

The data collected by snapshot receivers deployed on rabbits was limited by receiver failures. We conducted 29 field trials with unique rabbit-GPS receiver pairings (n = 20 individuals); 9 animals were re-fitted with different micro-GPS collars because of receiver malfunctions. Micro-GPS receivers were deployed multiple times over the study period. Deployment of snapshot GPS receivers (n = 29) functioned as expected in only 14% of cases, with malfunctions caused by premature battery failures (n = 18), software failures (n = 4), and unknown causes (n = 3). Snapshot receivers were deployed on animals for an average of 24 ± 1.8 days (range = 5–35), but GPS data were acquired for a mean of only 9 ± 1.6 days per animal (range = 0–35). The receivers were programmed to attempt 1 fix every 15 min, and in total, they collected 17,723 3-D fixes. Excluding receivers that failed to obtain even a single fix (n = 7), overall 3-D FSR from snapshot receivers on animals averaged 82.0 ± 4.2%. Snapshot receivers used a mean of 8.6 ± 0.02 satellites to estimate GPS locations with an average HDOP of 6.3 ± 0.01.

Traditional micro-GPS receivers operated more consistently than snapshot micro-GPS receivers during field trials with rabbits. We conducted 44 trials with unique rabbit-GPS receiver pairings using 35 individuals (six animals were collared with one or more different GPS receivers following malfunctions). Field trials with traditional GPS receivers were completed as planned in 59% of trials, with most malfunctions related to fatigue of wires connecting the battery and GPS circuit board (n = 14); this occurred only in the initial deployments and was eliminated with a design modification that fixed the battery in place on the collar to minimize friction between the wires connecting the battery and GPS circuit board. We also documented software failure (n = 6) and unknown or user error (n = 2). Traditional GPS receivers were deployed for an average of 14 ± 1.0 days (range = 1–36), and the GPS receivers logged location data for a mean of 11.0 ± 1.3 days (range = 0–36). Excluding receivers that failed to obtain a single fix (n = 6), overall FSR for all acquired fixes (2-D and 3-D) averaged 80.8 ± 2.1%, and overall 3-D FSR for traditional receivers was 72.4 ± 2.4%. Collecting 1 fix every 60 min, traditional receivers collected a total of 8,903 fixes, 90% of which were 3-D. The mean TTF for all fixes was 43.4 ± 0.2 s. Traditional receivers used on average 5.9 ± 0.02 satellites to estimate a location with a mean HDOP of 1.6 ± 0.02.

**Fig 5:**
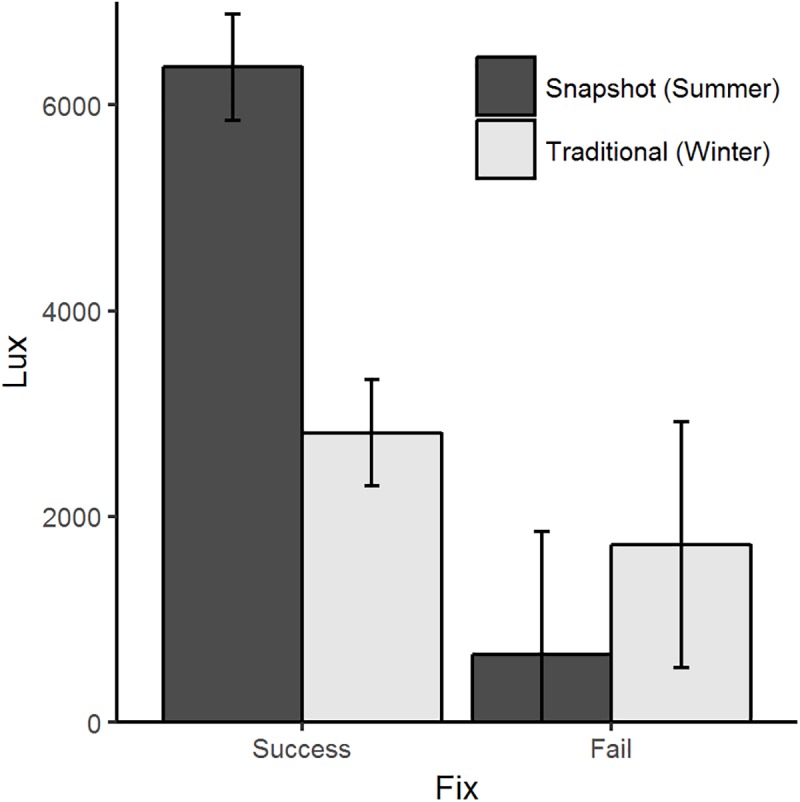
Mean (±SE) light values (lux) relative to micro-GPS receiver performance. When collars with both micro-GPS receivers and light loggers were deployed on free-ranging pygmy rabbits, successfully acquired locations (Success) had greater light levels than missed fixes (Fail).

**Fig 6:**
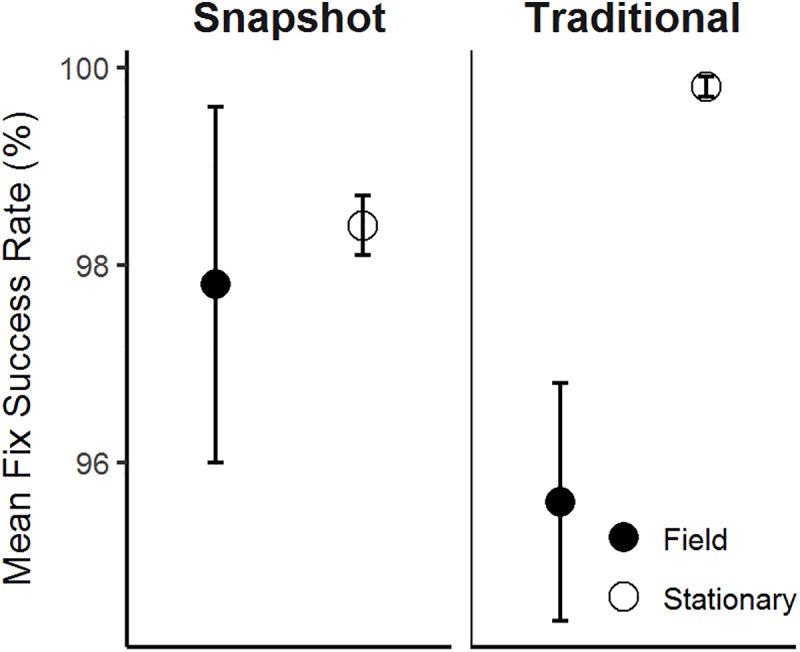
Fix success rates (±SE) of micro-GPS receivers deployed on free-ranging pygmy rabbits (field) deemed to be above ground and stationary tests (stationary). “Above ground” fix attempts were classified using a predetermined light threshold.

Data from light sensors used in conjunction with GPS receivers placed on rabbits suggested that burrow use, but not above-ground behavior, influenced FSR of the micro-GPS receivers. We collected a total of 4,779 fixes with paired data from light sensors for 18 rabbits during summer 2015 and winter 2016. During summer 2015, light sensors were paired with snapshot GPS receivers; during winter 2016, light sensors were paired with traditional GPS receivers, and consequently, we cannot independently interpret the influence of season and technology. Visual inspection of mean lux levels for missed and successful fixes suggested that lux levels for missed fixes were lower than mean lux levels for successful fixes during both seasons (Fig 5), indicating that missed fixes were at least partly attributable to burrow use. Interestingly, the lux levels for missed fixes were higher during winter, and we suspect that this might be influenced by use of subnivian tunnels by rabbits. We removed location data from animals estimated to be in a burrow using seasonal 95% cutoff values generated from light sensor performance tests (summer < 132 lux and winter < 538 lux). The threshold lux levels reflect seasonal differences in natural light, and the winter value was likely higher than summer because of light reflected off of snow and into burrow entrances. Contrary to our expectations, FSR from animals estimated to be above ground was comparable to the FSR for above-ground stationary tests, which suggests that animal behavior did not have a significant effect on FSR when rabbits were above ground (Fig 6).

## Discussion

Our study was the first to comprehensively quantify performance measures for micro-GPS telemetry receivers suitable for tracking mammals weighing < 1 kg. Micro-GPS receivers collected data with relatively high FSR and small LE values. Of the two types of micro-GPS receivers that we evaluated, traditional receivers produced more accurate locations than snapshot receivers (Fig 1). Both types of receivers performed poorly when in burrows, and to a lesser extent, when under shrub cover. Fix success rates acquired while animals wore the micro-GPS receivers above ground did not differ from locations acquired during stationary above-ground trials, suggesting that animal behavior other than burrowing did not markedly influence micro-GPS receiver performance in our study system. Our results suggest that micro-GPS receivers are capable of addressing questions about space use and resource selection by small mammals, but that additional techniques might be needed to identify use of habitat structures (e.g., burrows, tree cavities, rock crevices) that could affect micro-GPS performance and bias study results.

Somewhat unexpectedly, the magnitude of LE (8–16 m) and FSR (96–100%) that we documented for micro-GPS receivers during stationary tests were similar to those documented for much larger and heavier GPS telemetry receivers used for larger animals in other studies. Although performance reported in the literature for heavier, traditional GPS receivers varies considerably, reviews suggest typical values for FSR around 95% and LE from 10 to 30 m [8,35]. We found only one study that used snapshot GPS telemetry on a terrestrial species (lowland tapirs, *Tapirus terrestris*), and the authors reported similar LE and FSR of their large snapshot GPS receivers to the snapshot micro-GPS receivers used in this study [36]. In addition, the micro-GPS receivers that we evaluated generated FSR and LE values similar to other “lightweight” (120–125 g) traditional GPS receivers [12,24].

Large LE in locations acquired using the snapshot micro-GPS receivers were generated more often at the start of each trial, although traditional micro-GPS receivers collected consistently more accurate data throughout the tests (Fig 3). As expected, the increased TTF for the traditional micro-GPS receivers often allowed those receivers to detect and exploit satellites with more optimal geometry (HDOP), resulting in more accurate location estimates. Although snapshot GPS receivers cannot calculate their own locations, their performance unexpectedly improved over time during the stationary trials (Fig 3), suggesting that those receivers might retain some satellite information through almanac data (i.e., coarse data on the orbits of all GPS satellites). We suspect that the process of obtaining those data, however, might be prolonged due to the restricted durations of time searching for satellites. Removing snapshot data collected during the initial 9 hr of stationary tests eliminated 75% of locations with LE > 300 m. In contrast, traditional receivers collected similarly accurate data from the start of each test (Fig 3), likely as a result of more flexibility in the time allotted to detect and process satellite signals and retain satellite information [37]. Additionally, a weak cyclical relationship was apparent in the traditional GPS data and appeared to be associated with number of satellites and satellite geometry.

Burrow cover markedly influenced the performance of micro-GPS receivers. As predicted, receivers below ground were unable to digitize satellite signals because the sky was nearly completely obstructed, resulting in reduced FSR relative to above-ground test locations (Fig 4). Satellite acquisition is necessary to obtain location estimates, and accuracy increases with the number of satellites [26] and their spacing [12,24]. Locations collected while underground were subject to limited satellite view, resulting in locations collected with fewer satellites and suboptimal spacing. When a receiver did generate a location while below ground, it was significantly less accurate than the locations generated by the above-ground counterparts (Fig 4). Previous work has used light-sensitive radio-transmitters [38], temperature loggers [39], and direct observations [40] to identify use of microhabitat features by small mammals. When tracking burrowing animals, biologists will require additional methods to screen location data, including use of additional features such as light sensors or activity sensors, because missed fixes and large location errors can introduce bias into habitat selection studies [8].

The influence of vegetation on micro-GPS performance differed between locations with and without burrow cover. Our original model results, which included the strong effect of burrows, suggested that vegetation characteristics had negligible overall influence on FSR and LE for both types of GPS receivers. Analyses of above-ground trials reaffirmed that shrub cover had limited influence on FSR for snapshot GPS receivers, however, LE was significantly influenced by increased surrounding canopy cover, although the influence was modest. In contrast, for traditional GPS receivers, analyses of only the above-ground data indicated significant, although modest, influence of vegetation on both FSR and LE. The minimal influence of shrub characteristics on traditional receivers confirms the assertion by Recio et al. [24] that low vegetation cover is unlikely to significantly affect FSR and LE. Micro-GPS receivers deployed on free-ranging rabbits in our study exhibited high FSR (81%), comparable to results reported for traditional micro-GPS receivers (13 g) deployed on European hedgehogs (85%) in open habitats [4], and higher than the 66% FSR reported for traditional GPS receivers (42–52 g) deployed on American martens (*Martes martes*; [37], which live in heavily forested landscapes. These results support the contention that sagebrush shrub cover had minimal influence on FSR.

In contrast to previous studies on larger mammals, performance of GPS receivers in our study was not markedly influenced by behavior when rabbits were above ground. Research conducted on larger species such as grizzly bears (*Ursus arctos*; [20], moose *(Alces alces*; [16], and white-tailed deer (*Odocoileus virginianus*; [41] documented reduced FSR when animals wore traditional GPS collars relative to stationary trials, suggesting that an animal’s activity and movement, such as bedding, can reduce FSR by altering the position or orientation of the collar [15,16,41]. However, positioning of a GPS collar and body for animals like rabbits may not change much across behaviors like feeding and resting. This difference and the fact that the sagebrush steppe vegetation is open relative to the forested landscapes used by the larger species, could have contributed to the higher fix success documented in our study system.

Our study compared two types of GPS receivers and evaluated the influence of microtopography, vegetation, and satellite geometry on performance. Although previous research has noted differences in performance of GPS receivers among manufacturers [9,10,42], neither ATS nor Telemetry Solutions had an equivalent micro-GPS using the alternate technology to compare performance. Functioning of snapshot GPS receivers in our study was highly variable and unpredictable due to battery malfunctions and software problems, although the quality of the location data was similarly accurate to traditional receivers.

Our trials provide data that researchers can use to predict the influence of performance of micro-GPS receivers on studies of space use and habitat selection by small mammals. Large LE values could mask fine-scale patterns in foraging behavior and movement, especially when an animal’s typical movements are small [43] or when an animal occupies a highly heterogeneous environment [18]. Because small, herbivorous mammals often exploit smaller home ranges [44] or disperse shorter distances [45] than larger species, assessing the magnitude of LE of micro-GPS receivers is especially important for small mammal research. For example, annual home ranges for pygmy rabbits are typically < 5 ha [46], orders of magnitude smaller than those of larger, more mobile species that have been the focus of most research using GPS telemetry (e.g., feral cats, *Felis catus*, 270 ha; [47] or moose,1200 ha; [48]). Location error can limit the research questions that can be addressed. For instance, at the Cedar Gulch study site, habitat patches associated with mima mounds are on average 11 m in diameter and separated by 30–50 m. If LE was > 20 m, a GPS collared animal located at the edge of one patch, might appear to be using resources in a neighboring patch, resulting in inaccurate estimates of resource use and habitat selection. Our work suggests that micro-GPS telemetry can be used to address fine-scale questions, but an understanding of the heterogeneity of the landscape and animal behavior is necessary.

For burrowing and non-burrowing small mammals, traditional and snapshot GPS-receivers have the ability to produce location data suitable for investigating resource selection and space use. Most large errors that we detected were a result of microhabitat, such as burrows, indicating that small mammals’ use of these types of features (e.g., burrows, tree cavities, or rock crevices) might affect the accuracy and subsequent analysis of GPS locations. However, micro-GPS receivers had low FSR below ground, so large location errors as a result of burrow use would be minimized by virtue of the fact that the devices would fail to collect locations. Because other taxa also exploit microhabitat features that will influence GPS performance, this information is useful for applications of GPS telemetry to other vertebrates, including birds and reptiles that also exhibit a right-skewed distribution of body sizes with a prevalence of small species [49,50]. Error could be reduced further for species that use burrows or other cavities by incorporating light or activity sensors to facilitate screening locations when animals occupied such structures. Although we did not test the use of accelerometers and micro-GPS performance, the traditional receivers used in this project incorporated accelerometers that could be used in conjunction with the GPS receiver to attempt a fix only after the receiver surpassed a predetermined movement threshold. Accelerometer-informed GPS data might be beneficial for small species that exploit microhabitat features for resting behaviors by restricting GPS receiver fix attempts at times when the receiver is unlikely to obtain a fix or to generate inaccurate location estimates [37,51]. As receivers improve, snapshot technology could minimize the tradeoff between fix interval and battery life, allowing for more fine-scale evaluation of small mammal movement, activity, and resource selection.
